# Study of the Compatibilization Effect of Different Reactive Agents in PHB/Natural Fiber-Based Composites

**DOI:** 10.3390/polym12091967

**Published:** 2020-08-30

**Authors:** Estefanía Lidón Sánchez-Safont, Abdulaziz Aldureid, José María Lagarón, Luis Cabedo, José Gámez-Pérez

**Affiliations:** 1 Polymers and Advanced Materials Group (PIMA), Universitat Jaume I (UJI), Av. Vicent Sos Baynat s/n, 12071 Castelló de la Plana, Spain; esafont@uji.es (E.L.S.-S.); aldureid@uji.es (A.A.); lcabedo@uji.es (L.C.); 2 Novel Materials and Nanotechnology Group, Institute of Agrochemistry and Food Technology (IATA), Spanish National Research Council (CSIC), Calle Catedrático Agustín Escardino Benlloch 7, 46980 Paterna, Spain; lagaron@iata.csic.es

**Keywords:** PHB, natural fiber, compatibilizer, cellulose, biocomposite

## Abstract

Fiber–matrix interfacial adhesion is one of the key factors governing the final properties of natural fiber-based polymer composites. In this work, four extrusion reactive agents were tested as potential compatibilizers in polyhydroxylbutyrate (PHB)/cellulose composites: dicumyl peroxide (DCP), hexamethylene diisocyanate (HMDI), resorcinol diglycidyl ether (RDGE), and triglycidyl isocyanurate (TGIC). The influence of the fibers and the different reactive agents on the mechanical properties, physical aging, and crystallization behavior were assessed. To evaluate the compatibilization effectiveness of each reactive agent, highly purified commercial cellulose fibers (TC90) were used as reference filler. Then, the influence of fiber purity on the compatibilization effect of the reactive agent HMDI was evaluated using untreated (U_RH) and chemically purified (T_RH) rice husk fibers, comparing the results with the ones using TC90 fibers. The results show that reactive agents interact with the polymer matrix at different levels, but all compositions showed a drastic embrittlement due to the aging of PHB. No clear compatibilization effect was found using DCP, RDGE, or TGIC reactive agents. On the other hand, the fiber–polymer interfacial adhesion was enhanced with HMDI. The purity of the fiber played an important role in the effectiveness of HMDI as a compatibilizer, since composites with highly purified fibers showed the greatest improvements in tensile strength and the most favorable morphology. None of the reactive agents negatively affected the compostability of PHB. Finally, thermoformed trays with good mold reproducibility were successfully obtained for PHB/T_RH/HMDI composition.

## 1. Introduction

The development of biobased biodegradable thermoplastic materials is a topic research of special interest because it can represent a cost-effective and environmental-friendly alternative to commodities [1]. Among the different biopolymers, polyhydroxylbutyrate (PHB), a bacterial origin biopolyester from the polyhydroxyalcanoates family (PHAs), has attracted a lot of attention. The applicability fields where the PHB-based material results are more interesting are those in which biodegradability is desired either because composting could be a viable option for their waste management or because they can potentially end up in the environment. Among those applications, we can highlight food packaging or disposable products such as single-use tableware, hygiene-related single-use products, straws, etc. [1,2,3,4]. The main strengths of the PHB that make it suitable for this type of application are its natural origin, its biodegradability, the absence of toxicity, and the high service temperature [5]. Indeed, PHB presents mechanical properties in terms of a stiffness and strength that is similar to PP, good barrier properties, which are comparable or even superior to PET [6,7,8,9,10], and it is biodegradable in different environments, such as soil and marine [7,11,12], and compostable at lab-scale, industrial, and home composting conditions [13]. 

However, PHB presents some shortcomings that limit its industrial applicability. PHB is a semicrystalline polymer that is capable of a high degree of crystallinity but has a relatively low crystallization rate. Hence, PHB suffers an appreciable embrittlement with time due to secondary crystallization and physical aging [14,15,16,17], and its long-term mechanical properties are characterized by low ductility and toughness. Indeed, the processing temperature window of PHB is very narrow: the lower limit is relatively high due to its high crystallinity, and the upper limit is relatively low because of its poor thermal stability in molten state (the degradation temperature is close to the melting temperature [7]). Altogether, these factors make PHB quite difficult to process, especially in the case of thermoforming [18]. In addition, one of the main limiting factors is its current high price. In this sense, the development of PHB-based composites using lignocellulosic fibers as fillers could contribute to a large extent to overcome the cost drawback maintaining the biodegradability and even improving the mechanical performance of PHB, allowing the valorization of vegetal wastes contributing to the circular economy.

Lignocellulosic fibers are hydrophilic materials composed by bundles of cellulose fibers embedded in a matrix of other non-cellulosic materials such as lignin, hemicelluloses, pectin, waxes, and other minor components [19]. The advantages of use lignocellulosic fibers as fillers are their availability, low cost, biodegradability, low density, high stiffness, and acceptable specific strength [20]. However, they also present shortcomings related to their thermal sensitivity and hydrophilic nature. In addition, depending on the vegetal source and/or the plant location and time of harvest, the composition, properties, morphology, and surface characteristics of different lignocellulosic fibers may differ significantly [21].

It is well known that the resultant properties of fiber-based composites depend not only on the properties of the constituents but are also determined by the fiber–matrix adhesion. The hydrophilic nature of the lignocellulosic fibers lowers the compatibility with the hydrophobic polymer. Nevertheless, according to Bhardwaj et al. [22], the relatively polar nature and presence of carbonyl groups (–C=O) in PHB as compared with other nonpolar matrices such as PP might cause a hydrogen-bonding-type interaction with the cellulosic fibers and relative better compatibility, as it has been also noticed by others in PHA/lignocellulosic composites [23,24]. However, these interactions are not enough to provide strong adhesion of PHB with lignocellulosic fibers, as it has been shown previously in PHA-based composites, which are filled with untreated lignocellulosic fibers [25,26,27]. Thus, the enhancement of fiber–matrix adhesion may be a key factor to exploit the full capabilities of these composites. 

Some attempts to improve interfacial adhesion are physical treatments (plasma or corona discharge), chemical purification treatments (dewaxing and delignifying treatments) of the fibers, grafting, or the use of additives such as compatibilizers or coupling agents [19,28,29]. Reactive compatibilization is an interesting cost-effective one-step strategy consisting of the use of small amounts of reactive agents that possess functional groups with a tendency to react with the –OH groups of the fibers and with the carboxylic end groups from polyesters by covalent bond interactions. Thus, the most popular reactive agents used include maleic anhydride groups, epoxy groups, or isocyanate groups [30,31]. Several examples of the use of reactive agents in polyester/fiber-based composites can be found in the literature. Diisocyanates have been used in PHBV/bamboo fibers [32] or poly(3-hydroxybutyrate-co-3-hydroxyvalerate) (PHBV)/poly(butylene adipate-co-terephthalate) (PBAT) /Switchgrass systems [33]. Epoxy-based reactive agents have been used in PLA/sisal fiber composites [34,35].

Another strategy could be by using radical generators that could arouse random linkages between the matrix and the reinforcement via radical intermediate species, such as peroxides. Dycumil peroxide (DCP) has been used to compatibilize PHBV/Miscanthus fiber composites [31] or PHB and PHBV/α-cellulose composites [36]. 

**Figure 1:**
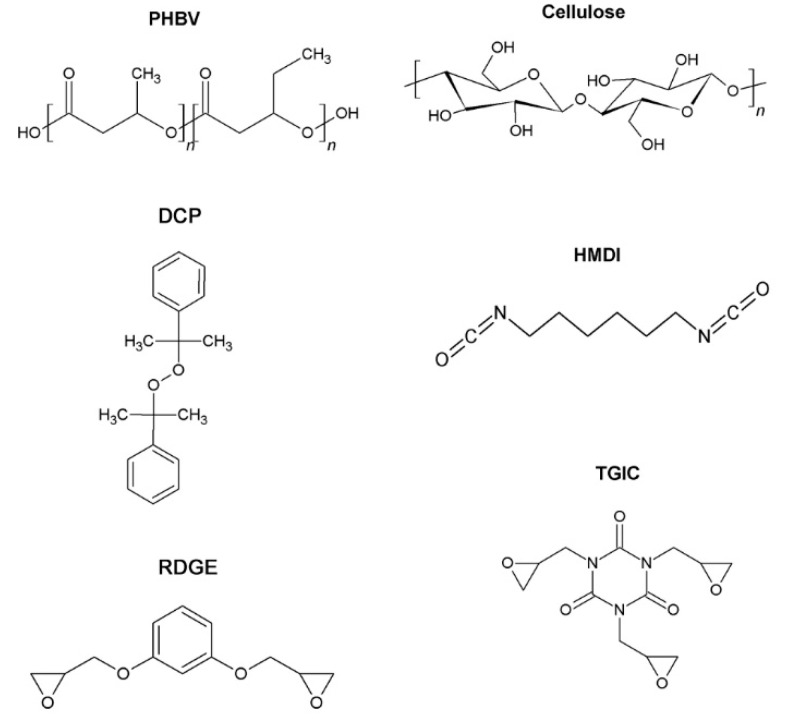
Chemical structures of polyhydroxylbutyrate (PHB), cellulose, and the reactive agents.

In this work, the efficiency as compatiblizers of four different reactive agents in fiber-based PHB composites was tested. The reactive agents used were dicumyl peroxide (DCP), hexamethylene diisocyanate (HMDI), resorcinol diglycidyl ether (RDGE), and tryglicidyl isocyanurate (TGIC). The chemical structures of them are shown in Figure 1. In order to reduce variables and better understand the role of each reactive agent in this study, a high purified commercial cellulose fiber (TC90) with an α-cellulose content >99.5 % was selected, being the filler load set at 10 phr (i.e., per hundred mass of resin) for all compositions. 

The effect of the different reactive agents on the PHB/cellulose interfacial interactions was studied by scanning electron microscopy (SEM), tensile tests, and dynamic mechanical analysis (DMA). Indeed, the effect of aging was assessed for all compositions. As maintained biodegradability is an important requirement for the applicability of these systems, the effect of the different reactive agents on the biodisintegration under standard composting conditions (ISO 20200) was also evaluated. 

With the aim of analyzing the influence of fiber purity on the compatibilization efficiency, untreated rice fibers (U_RH) and chemically purified rice husk fibers (T_RH) according to a previous work [37] were used using HMDI as a compatibilizer. The mechanical performance and the morphology were analyzed, and the results were compared with the use of the commercial cellulose. 

Finally, since packaging is one of the potential application fields for these composites, the suitability of PHB/T_RH/HMDI composites to be processed by thermoforming was tested. This process has been chosen for both its difficulty and for being one of the most popular forming techniques used in the packaging industry.

## 2. Materials and Methods 

### 2.1. Materials

Poly(3-hydroxybutyrate) was supplied by Biomer^®^ (Schwalbach, Germany) in pellet form (P309). Purified alpha-cellulose fiber grade with an alpha-cellulose content >99.5% (TC90) was purchased from CreaFill Fibers Corp. (Chestertown, MD, USA). Rice husk (RH) by-product from the rice production process was kindly provided by Herba Ingredients (Valencia, Spain). The four reactive agents used (dicumyl peroxide (DCP), hexamethylene diisocyanate (HMDI), resorcinol diglycidyl ether (RDGE), and triglycidyl isocyanurate (TGIC)) were purchased from Sigma Aldrich (Madrid, Spain). Sodium hydroxide (NaOH, 98%), hydrogen peroxide (H_2_O_2_, 30%), glacial acetic acid (CH_3_COOH, 99%), and sulfuric acid (H_2_SO_4_, 98%) were purchased from Sigma Aldrich (Madrid, Spain).

### 2.2. Rice Husk Fibers Preparation

RH fibers were ground in a mechanical knife mill and then sieved in 140 µm mesh. These untreated RH fibers were named U_RH. A fraction of the ground and sieved RH fibers were subjected to a two-stage purification treatment in order to remove the major parts of impurities and non-cellulosic components such as waxes, lignin, and hemicelluloses. The first stage consisted of an alkaline attack with NaOH (5% *wt/v*, fiber/liquid ratio of 1:20, 80 °C, 2 h). This treatment was applied twice. The second stage consisted of an oxidative attack with peracetic acid (PAA) (fiber/liquid ratio of 1:20, 80 °C, 4 h). The peracetic acid was prepared by the mixing of 30%(*v/v*) hydrogen peroxide and acetic acid in the reaction medium with a volume ratio of 3:1 at room temperature and 1% (*w/w*) of sulfuric acid as catalyzer. This procedure was adapted from the literature [38,39]. After each stage, the fibers were filtered and washed repeatedly in distilled water until neutral pH was reached. The purified powder was dried at 60 °C for at least 24 hours and ground again to break the aggregates formed during the filtration process and then sieved in a 140 µm mesh. The as-treated RH fibers were named as T_RH. 

### 2.3. Composites Preparation

In order to assess the role of reactive agents as compatibilizers, compounds of purified commercial cellulose (TC90) were prepared with all reactive agents. The effect of the cellulose purity was studied on rice husk fibers, with and without chemical treatment, using HMDI as the reactive agent. For the sake of comparison and to evaluate the effects of the compatibilizers on the matrix, blank compounds (without cellulose) were prepared as controls. All the compositions studied are summarized in Table 1. 

The compounds were prepared by melt extrusion in a twin-screw co-rotating extruder (DUPRA SL, Castalla, Spain) with an L/D ratio of 24 and a diameter of 2.5 cm. All the components were dried before extrusion; PHB pellets were dried in a dehumidifier Piovan DPA50 (Piovan, Maria di Sala VE, Italy) at 60 °C following the producer’s drying recommendations and the fibers (TC90, U_RH and T_RH) were dried in an oven at 100 °C for at least 2 h. The formulations were manually premixed in zip-bags. The temperature profile of the extruder was set as follows: 165/170/175/180 °C (from the hopper to the extruder die), and the screw speed was kept constant at 40 rpm. The extrudate material was pelletized and dried following the same considerations as pure PHB.

From the extruded pellets, different samples were obtained by compression molding in a parallel plate hot-press (180 °C, 2 min for premelting followed by 2 min at 3 bar): bars of 50 × 12.5 × 3.5 mm for dynamic mechanical analysis tests, films of 0.4 mm nominal thickness for uniaxial mechanical tests, films of 0.2 mm nominal thickness for composting tests, and films of 0.8 mm nominal thickness for thermoforming essays. Samples with neat PHB were processed and tested at the same conditions as the compounds.

### 2.4. Methods

The morphology of PHB/TC90, PHB/U_RH, and PHB/T_RH composites with and without reactive agents was examined by scanning electron microscopy (SEM), using a high-resolution field-emission microscope (JEOL 7001F, Tokyo, Japan). The samples were prepared by cryofracturing after immersion in liquid nitrogen and then coated by sputtering with a thin layer of Pt. 

Differential scanning calorimetry (DSC) experiments were conducted on a DSC2 (Mettler Toledo, Columbus, OH, USA) with an intracooler Julabo FT900 (Julabo, Seelbach, Germany) calibrated with Indium standard before use. Samples were analyzed at 0 days (after hot-pressed films obtention) and after 100 days, to account for physical aging at room temperature. The samples weighing typically 6 mg were first heated from −20 °C to 200 °C at 10 °C/min, kept for 5 min to erase thermal history, and cooled down to −20 °C at 10 °C/min. Then, a second heating scan to 200 °C at 10 °C/min was performed. Crystallization temperatures (T_c_), melting temperatures (T_m_), and melting enthalpies (ΔH_m_) were calculated from all respective heating/cooling scans. The crystallinity (X_c_) of the PHB–reactive agent compositions was determined by applying the expression (1) [40]:

(1) $$X_{c}\left(\%\right)=\frac{{\Delta H}_{m}}{w\cdot {\;\Delta H}_{m}^{0}}\times 100$$

where ΔH_m_ (J/g) is the melting enthalpy of the polymer matrix, ΔH°_m_ is the melting enthalpy of 100% crystalline PHB (perfect crystal) (146 J/g) [16], and w is the PHB weight fraction in the blend. 

Tensile tests were conducted in a universal testing machine Shimatzu AGS-X 500N (Shimatzu, Kyoto, Japan) at room temperature with a crosshead speed of 10 mm/min. Dumbbell 400 µm-thick samples were die-cut from the hot-pressed films and tested according to ASTM D638 (Type IV) standard. The samples were tested immediately after processing (0 days) and after 15 days of aging at room temperature. All the samples were stored in a vacuum desiccator at ambient temperature until tested.

Dynamic mechanical analysis (DMA) experiments were conducted on hot-pressed sample bars (55 × 12.5 × 3.5 mm) in an AR G2 oscillatory rheometer (TA Instruments, New Castle, DE, USA) equipped with a clamp system for solid samples (torsion mode). Samples were heated from −20 °C to melting temperature with a heating rate of 2 °C/min at a constant frequency of 1 Hz. The maximum deformation (γ) was set to 0.1%. 

Disintegration tests under standard composting conditions (ISO 20200 [41]) were carried out with samples of (15 × 15 × 0.2 mm^3^) obtained from hot-pressed plates. Solid synthetic waste was prepared by mixing 10% of activated mature compost (VIGORHUMUS H-00, purchased from Burás Profesional, S.A., Girona, Spain), 40% sawdust, 30% rabbit feed, 10% corn starch, 5% sugar, 4% corn seed oil, and 1% urea. The water content of the mixture was adjusted to 55%. The samples were placed inside mesh bags to simplify their extraction and allow the contact of the compost with the specimens; then, they were buried in compost bioreactors at 4–6 cm depth. Bioreactors were incubated at 58 °C. The aerobic conditions were guaranteed by mixing the synthetic waste periodically and adding water according to the standard requirements. Two replicates of each sample were removed from the boxes at different composting times for analysis. Samples were washed with water and dried under vacuum at 40 °C until reaching a constant mass. The disintegration degree was calculated by normalizing the sample weight to the initial weight with Equation (2):

(2) $$D=\frac{m_{i}-m_{f}}{m_{i}}\times 100$$

where m_i_ is the initial dry mass of the test material and m_f_ is the dry mass of the test material recovered at different incubation stages. The disintegration study was completed taking photographs for visual evaluation.

The thermoformability of PHB/T_RH/HMDI was tested by a vacuum-assisted thermoforming technique in a pilot plant (SB 53c, Illig, Helmut Roegele, Heilbronn, Germany) equipped with an infrared emitter heating device. The mold used was a female circular tray that was 55 mm in diameter and 15 mm in depth with an edge radium of 5 mm. Rectangular hot-pressed sheets of a typical thickness of 800 μm were used for this study. The sheets were stamped with a square grid pattern (0.5 × 0.5 cm) in order to track the deformation that occurred during their mold conformation. The infrared heater was set to 600 °C, whereas the heating and vacuum times (ranging between 20–45 s and 3–20 s, respectively) were optimized in each case to obtain the best results. 

## 3. Results

### 3.1. Influence of Reactive Agents in PHB/Cellulose Composites

#### 3.1.1. Morphological Analysis

**Figure 2:**
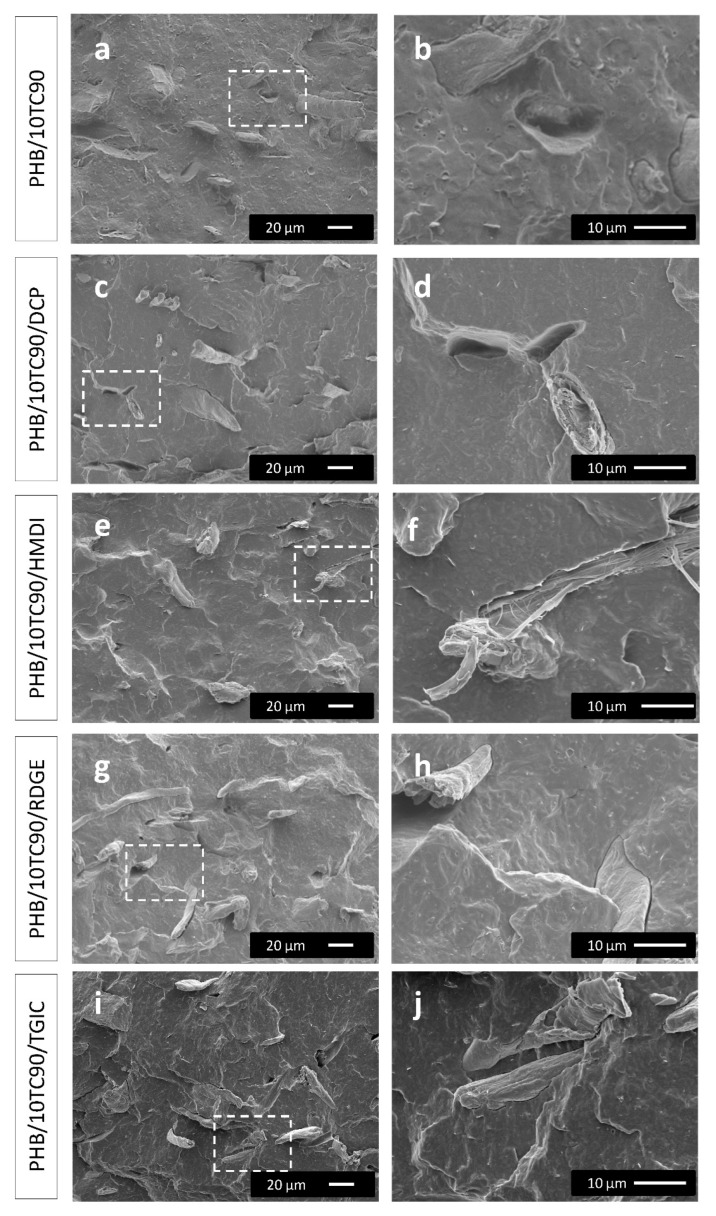
SEM micrographs of PHB/TC90 composites with and without reactive agents. The images in the right column (**b**,**d**,**f**,**h**,**j**) show higher magnifications of the areas indicated with a square in their corresponding images in the left column (**a**,**c**,**e**,**g**,**i**).

In order to assess the role of the reactive agents, blends with TC90 were prepared as detailed in the experimental section. The morphology of the PHB/TC90 composites with and without the reactive agents has been analyzed by SEM. Low magnification images were used to study the distribution of the fibers within the polymer matrix, and high magnification ones were used to examine the fiber/matrix interface. The micrographs of the different composites are shown in Figure 2.

As it can be observed in Figure 2a,c,e,g,i, in general, the fibers are well distributed within the polymer matrix, and we do not detect the presence of fiber aggregates, indicating an effective compounding. Despite this well dispersed and distributed morphology points to some type of fiber/matrix interaction (probably hydrogen bonding), the presence of some voids and prints caused by detached fibers (Figure 2a) as well as the gap observed between the fiber and the matrix (Figure 2b) are indicative of a certain lack of adhesion. Then, it can be said that there are some interactions in the melt that favor homogeneous dispersion, but those are not strong enough to provide an effective interface between both components. 

Regarding the reactive agents, no remarkable differences in morphology are detected in PHB/TC90 composites with reactive agents compared with the composite without them. Although the major part of the fibers seems to be well embedded into the polymer matrix, some pull-out and detached fibers are detected, as well as a small gap between the fibers and the matrix. With regard to the use of DCP, contrary to other works reported in literature [31,36,42], in our case, no clear enhancement of compatibilization between fibers and matrix can be appreciated by SEM. In the same way, any compatibilization effect was found for the RDGE. Similarly, the TGIC did not show any additional compatibilization effect, as this was unexpected [34]. However, in PHB/TC90/HMDI composites, there is an improvement of fiber–matrix adhesion, finding no fiber pull-outs or detachment in the micrographs (Figure 2e). In this case, the fibers seem to be well covered by the polymer, and fibers broken on their longitudinal direction can be observed (Figure 2f), thus indicating a cohesive failure. Thus, SEM observations would be in agreement with a strong adhesion between the fibers and the PHB matrix. This is probably due to the formation of urethane linkages between the isocyanate groups of HMDI and hydroxyl (–OH) groups from the fibers and/or hydroxyl or carboxylic chain ends of PHB, as it has been proposed in the literature for biopolyester/fiber systems compatibilized with isocyanates [33].

#### 3.1.2. Thermal Properties

DSC experiments were run in all samples. The thermograms were obtained from the films recently processed (0 days) and after 100 days of storage at room temperature, when it is supposed that all secondary crystallization and physical aging phenomena has taken place [15,43]. It can be seen in the thermograms that aging only affected the first heating scans. The second heating scans, after erasing the thermal history and controlled cooling at 10 °C/min, were the same for 0 and 100 days, thus confirming that there were no significant structural changes during storage. Therefore, no signs of degradation were evidenced for this period. All the results are summarized in Table 2.

The melting behavior and crystallinity index during first heating scans of the composites are affected in different extents by the different components. However, it must be considered that the crystal morphologies developed during cooling correspond to processing conditions, which implies higher cooling rates with respect to DSC-controlled cooling at 10 °C/min.

Neat processed PHB presents a melting peak temperature of 175 °C and a crystallinity index (X_c_) of 49% at 0 days. After aging, X_c_ increases to 59%, and the melting peak temperature changes to 170 °C, due to secondary crystallization [44]. After erasing thermal history, the cooling of PHB yields a crystallization peak temperature of 117 °C and ΔH_c_ of 91 J/g, which corresponds to an X_c_ value of 63% (for either aged or unaged samples).

Regarding the influence of the reactive agents on X_c_, at 0 days, PHB/DCP and PHB/HMDI show similar crystallinity indexes than neat PHB, whereas in PHB/RDGE and PHB/TGIC compounds, X_c_ is slightly higher. The melting temperatures, on the other hand, are similar to that corresponding to neat PHB, with the exception of DCP, which is slightly inferior. After 100 days of aging, the crystallinity index of PHB with the different reactive agents is in all cases inferior with respect to neat PHB, especially in case of DCP, for which melting parameters remain practically unchanged compared to the unaged sample. This can be related with the crosslinking effect of DCP, which generates free radicals and disrupts the linearity of the PHB chains, thus limiting the maximum crystallinity that can be developed [45]. 

The presence of the reactive agents (with or without fibers) seems to partially hinder the secondary crystallization [46], as the crystallinity index achieved at 100 days is in all cases inferior to that corresponding to neat PHB. Indeed, it seems that the sole presence of the fibers restricts the mobility of polymer chains, partly hindering the development of crystallization during aging, since the increase in the crystallinity index is not observed over time for the PHB/TC90 composites. According to the literature, fillers can restrict the polymer chain mobility [24], which could result in a stabilized crystallinity index over time. 

After erasing the thermal history, no remarkable differences in crystallization temperatures or enthalpies were observed among the compounds, except in the case of HMDI addition. Compounds with HMDI showed lower T_c_ values than the other compositions, finding a reduction of T_c_ from 117 °C to 106 °C in PHB/HMDI and 111ºC in PHB/TC90/HMDI samples. These findings can be related with some hindered motion of the polymer chains, thus suggesting the interaction of HMDI with the polymer matrix and the fibers [47]. 

With respect to melting in second heating scans, after low cooling rates where polymer chains had enough time and mobility to develop high crystallinity (10 °C/min during DSC test conditions), the crystallinity indexes and melting temperatures of the different compositions were similar to those corresponding to neat PHB, except for the compositions containing DCP. For DCP-containing compounds, significant reductions of T_m_ and X_c_ was detected, being in agreement with some crosslinking of the PHB matrix with the peroxide initiator DCP [45]. 

#### 3.1.3. Mechanical Properties

**Figure 3:**
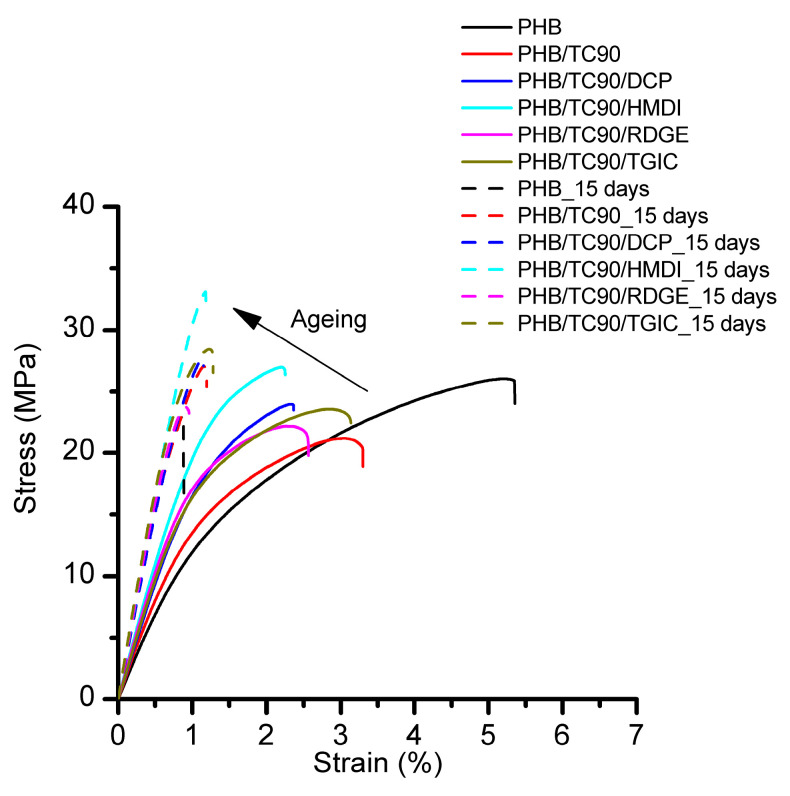
Representative stress–strain curves of neat PHB and PHB/TC90 composites at 0 days (solid lines) and after 15 days of aging (dashed lines).

**Figure 4:**
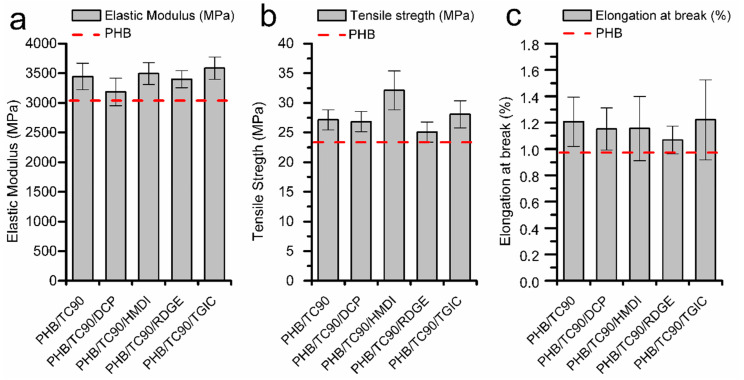
(**a**) Elastic modulus, (**b**) tensile strength, and (**c**) elongation at break of aged samples of neat PHB and PHB/TC90 composites with and without reactive agents.

PHB is known by its physical aging and secondary crystallization [15,16,43]. So, the mechanical performance depends on time after its processing. For such a reason, tensile properties have been assessed after processing (0 day) and after 15 days stored at room temperature. According to Corre et. al., after such a period of time, the variations on the mechanical properties are so small that it can be said that the properties are stabilized [16]. The mechanical properties of all compounds were determined by uniaxial tensile tests up to failure. Representative stress versus strain curves of the composites with TC90 are shown in Figure 3. The average parameters obtained from the curves are summarized in Table 3, and selected values are represented in Figure 4 to illustrate the trends observed. 

Neat PHB shows a high variation in its tensile behavior due to aging. The elastic modulus increased more than 100% after 15 days, and the deformation at break decreased five times. In both cases, the failure mode is brittle, with unstable fracture and without showing necking nor evidence of shear yielding. The cause for such behavior is attributed to secondary crystallization and the physical aging of the amorphous region [15,16,43].

When reactive agents are added to PHB, at 0 days, there is a minimum increase in elastic modulus in the case of addition of DCP, RDGE, and TGIC and a more pronounced one with HMDI (Table 3). However, after 15 days, PHB/DCP elastic modulus is 9% lower than neat PHB. The reactivity with the polymer matrix can account for this behavior. For DCP, the generation of some crosslinking is in agreement with lower development in crystallinity and hence a reduction on elastic modulus [45]. With respect to RDGE and TGIC, some reactivity is also evidenced, but in a way that it seems to affect the amorphous region, since after 15 days, they are able to withstand higher deformations prior to rupture and show higher tensile strength before a crack generates and propagates through the PHB. It could be hypothesized that more voluminous RDGE and TGIC disrupt the pseudo-order in the rigid amorphous phase region. This is not observed in PHB/HMDI compounds, probably because of the small and linear geometry of the HMDI molecule, which does not prevent the rearrangements of the amorphous phase that take place during physical aging [16,17,43,48].

When cellulose (TC90) is added to neat PHB (at 0 days), an increase in elastic modulus and a reduction of tensile strength and ductility is observed (Figure 3). The increase in modulus of elasticity is attributed to the reinforcement effect of the cellulose fibers. However, the addition of TC90 promotes rupture at even lower stress that in the case of Neat PHB (and therefore, much lower than yielding). Along with the increase of elastic modulus, this behavior suggest that cellulose acts as a reinforcement in PHB matrix at low strains, but after a certain point, it promotes the appearance of large defects that nucleate cracks that lead to brittle fracture, explaining the low values of tensile strength and elongation at break.

When reactive agents are added, the elastic modulus rises in all cases with respect to either cellulose without reactive agents or PHB with reactive agents. Similarly, the tensile strength also rises; both suggest an increase of affinity between the matrix and reinforcement. However, deformation at break does not increase, and it remains at a similar value as uncompatibilized TC90. 

To better understand the role of TC90 and the different reactive agents on the mechanical behavior of PHB after aging (15 days), the elastic modulus, tensile strength, and elongation at break values of the composites are depicted in Figure 4. 

After aging, the incorporation of TC90 fibers to PHB results in a slight improvement of elastic modulus and tensile strength, while showing a comparable elongation at break. In Figure 4a, it can be appreciated that the elastic modulus of all the composites is improved with respect to neat PHB (about 13% for PHB/TC90). This behavior can be reasonably ascribed to the affinity between the rigid cellulose fibers and the PHB matrix. The addition of the reactive agents to the PHB/TC90 compounds did not show any remarkable additional improvement of the elastic modulus at 15 days with respect to the composites without them. However, in the case of tensile strength values, an interesting increase of about 18% with respect to PHB/TC90 is detected in the PHB/TC90/HMDI compound. This rise suggests that HMDI had an effective compatibilizer role, strengthening the bonding of the cellulose fibers with the PHB matrix. 

**Figure 5:**
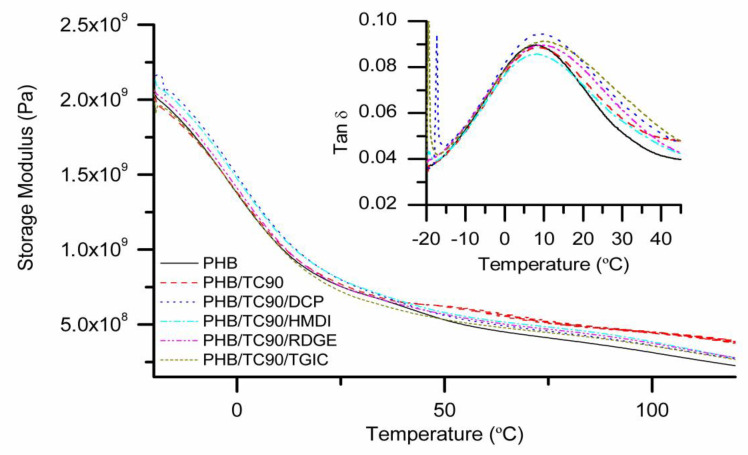
Storage modulus (G’) and tan δ (inset) evolution with temperature for neat PHB and PHB/TC90 composites with reactive agents.

The mechanical characterization of the composites was completed by DMA testing. Their storage modulus (G’) and the damping factor (tan δ) evolution with temperature are represented in Figure 5.

No appreciable differences in storage modulus among the different samples are observed. These results are in agreement with the elastic modulus observed in mechanical tests. The tan δ represents the energy dissipated during the dynamic tests, and the tan δ peak is usually used to determine glass transition (T_g_) in semicrystalline polymers [49]. In this case, all the samples present T_g_ values around 10 °C. Nevertheless, the tan δ versus temperature plot suggest certain restricted mobility of the polymer chains in composites, since the tan δ peaks are broader than neat PHB [50]. Moreover, the height of the tan δ peak corresponding to PHB/TC90/HMDI is reduced compared to the rest of composites, indicating the further hindered motion of the polymer chains. This can be related with a better interaction between PHB and TC90 fibers due to the compatibilization effect of HMDI [30]. 

### 3.2. Influence of Fiber Purity

#### 3.2.1. Morphological Analysis

As it has been discussed above, HMDI has demonstrated its efficiency at improving the interfacial adhesion between PHB and highly purified commercial cellulose fibers (TC90). In this section, the compatibilization ability of HMDI is tested using an unpurified rice husk fiber (U_RH) and a chemically treated rice husk fiber (T_RH) to study the combined effect of purification treatment and compatibilizer on the interfacial PHB/fiber interactions. 

**Figure 6:**
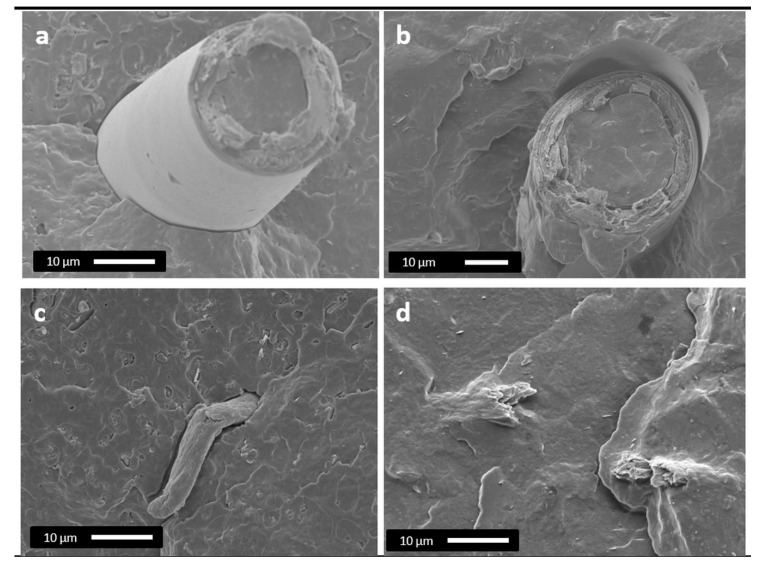
(**a**) SEM micrographs of PHB/U_RH, (**b**) PHB/U_RH/HMDI, (**c**) PHB/T_RH, and (**d**) PHB/T_RH/HMDI.

SEM micrographs (shown in Figure 6) allow visualizing qualitatively the matrix/fiber interface interactions of PHB/ U_RH and PHB/T_RH composites, with and without HMDI.

As it can be observed in Figure 6a, U_RH fibers within the compound present a smooth surface and show a gap between the fiber and the polymer matrix. A similar gap between these two components is also observed in PHB/U_RH/HMDI composition (Figure 6b). In case of treated fibers (PHB/T_RH), the fibers present a smaller diameter and a rougher surface than the untreated ones, but detachment of the fibers is also detected, thus indicating a certain lack of adhesion (Figure 6c). In the case of treated and compatibilized fibers (Figure 6d), they appear well covered by the polymer and no gap or signs of detachment at the interphase are detected, suggesting an improved adhesion.

#### 3.2.2. Mechanical Properties

**Figure 7:**
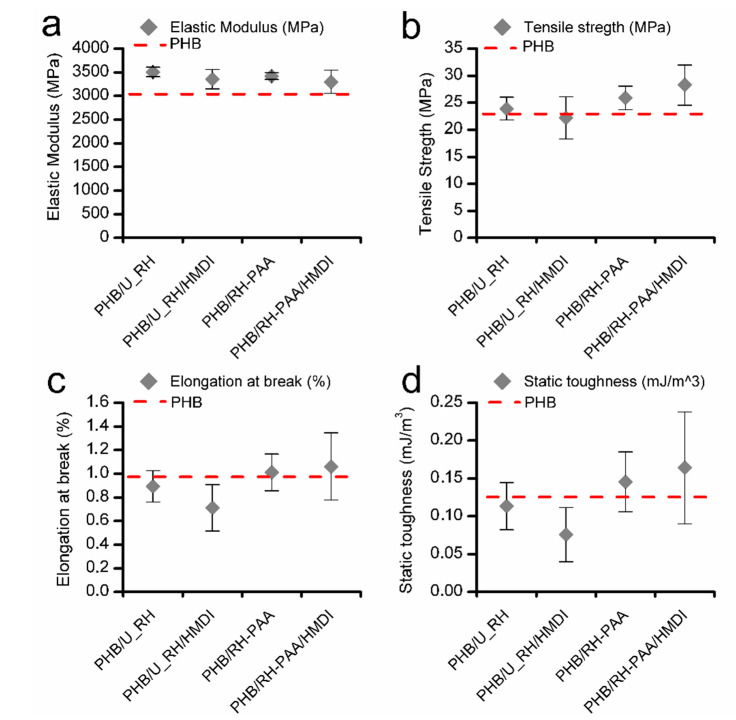
(**a**) Elastic modulus, (**b**) tensile strength, (**c**) elongation at break, and (**d**) static toughness of neat PHB, PHB/U_RH, and PHB/T_RH composites with and without reactive agents.

Uniaxial tensile tests of PHB/U_RH and PHB/T_RH with and without HMDI were performed on 15-day aged samples at room temperature. Elastic modulus, tensile strength, elongation at break, and the static toughness obtained from the area below the stress–strain representative curves for each composite are depicted in Figure 7. The mechanical parameters corresponding to neat PHB are also represented as a reference. 

The addition of either U_RH or T_RH fibers produces a reinforcement effect with respect to neat PHB, since an increment of the modulus of elasticity is detected in all cases. On the other hand, the tensile strength seems to increase only when the treated fibers are the ones added, where the use of HMDI on such treated fibers produced an additional improvement of this parameter. For composites with treated fibers, the elongation at break remains simmilar to that of neat PHB, whereas for the untreated ones, this parameter is reduced (especially in the case with HMDI, in agreement with values reported for PHB/HMDI samples in Table 3). These results suggest that in the case of the untreated fibers, there is no interaction of HMDI between the polymer and the reinforcement. 

Despite the fact that in all cases, the samples present a brittle behavior, the addition of the treated fibers leads to a visible trend of improvement of the static toughnes, compared with neat PHB, especially with the addition of HMDI. Nevertheless, the enhancement of the mechanical performance was not as pronounced as in the case of the compound prepared in the previous section, with high-purity commercial cellulose (PHB/TC90/HMDI).

#### 3.2.3. Thermoforming Ability

**Figure 8:**
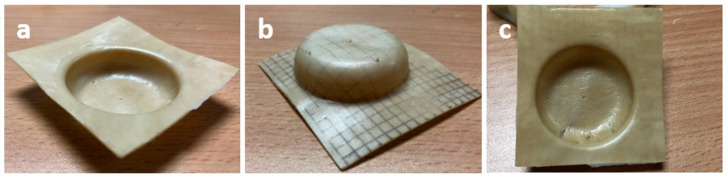
PHB/T_RH/HMDI thermoformed tray: (**a**) bottom-side view, (**b**) top-side view and (**c**) bottom view.

PHB-based composites reinforced with fibers are considered an attractive alternative to commodities for short-term life applications such as packaging. For this reason, it is of particular interest to test their processability by thermoforming, which is a conventional technique that is usually applied in this industrial field. In this regard, PHB/T_RH/HMDI films have been thermoformed into trays following the procedure described in the experimental section, and the best results obtained are presented in Figure 8.

As it can be observed in Figure 8, thermoformed trays with good mold reproducibility and relatively good thickness distribution (punctual thickening is observed in Figure 8c) can be obtained by adjusting the operational parameters. In our case, the best results were obtained for a heat resistance temperature fixed at 600 °C, a heating time of 35 s, and 7 s applying vacuum. The difficulties of thermoforming semicrystalline polymers [18,51,52] and even more filled with fibers are well recognized, so the results obtained here are very promising.

#### 3.2.4. Biodisintegration in Composting Conditions

**Figure 9:**
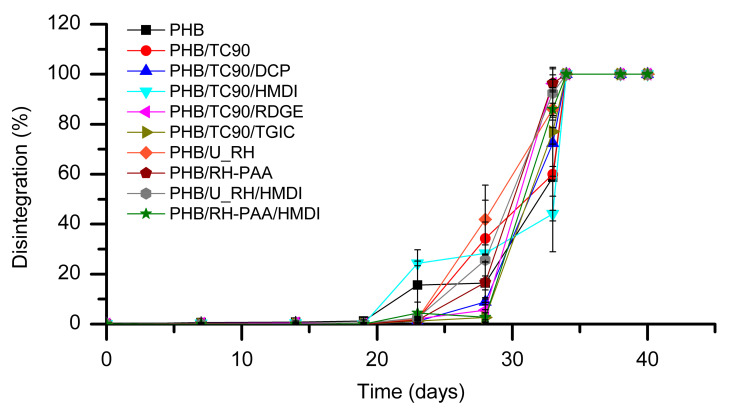
Disintegration of neat PHB and the studied composites over time under standard composting conditions (ISO 20200).

**Figure 10:**
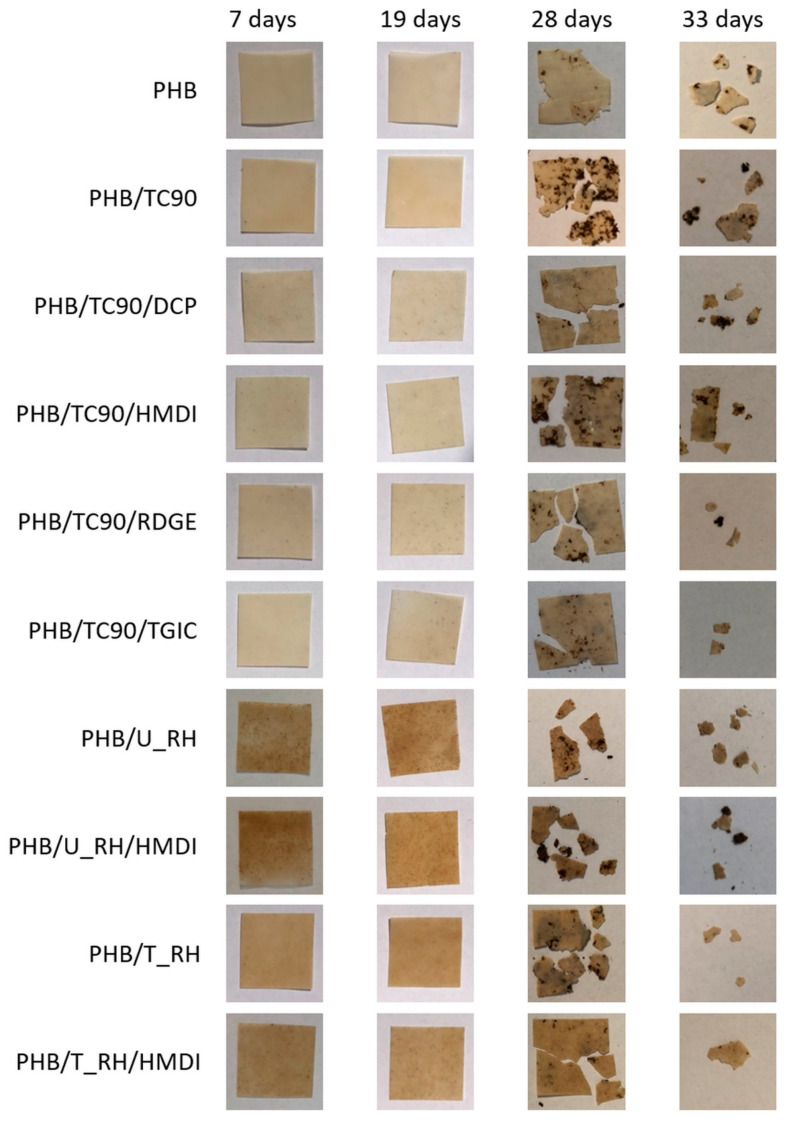
Pictures of neat PHB and the studied composites at different composting times.

One of the strengths of the PHB/fiber composites that make them especially attractive for packaging applications is their biodegradability and specifically their compostability. For this reason, it is of special interest to evaluate the effect of the reactive agents on the behavior under normalized composting conditions of the composites. Biodisintegration tests were conducted according to the ISO 20200 standard. The weight loss over time of the tested materials is represented in Figure 9, and pictures of the samples at different composting times are depicted in Figure 10. 

As shown in Figure 9, in neat PHB, the biodisintegration process occurs with an incubation period of about 23 days. From this time, appreciable weight loss is detected, and total disintegration (considered when fragments >2 mm are not detectable) is reached at 35 days of composting. For PHB/TC90 composites containing DCP, RDGE, and TGIC, the incubation period seems to be slightly longer. At intermediate composting times, some differences in weight loss among the samples were detected. However, these differences do not reveal a clear trend related with the presence or not of the different fibers or reactive agents. In any case, all the compositions reached total biodisintegration in the same period than neat PHB (35 days). Some authors have reported an accelerated biodegradation of biopolyesters with the incorporation of lignocellulosic fibers [23,25]. In our case, no remarkable differences were observed, as we had already previously noticed in PHBV/TC90 [53] and PHB/lignocellulosic composites [54]. 

As shown in Figure 10, no remarkable physical changes can be visually detected in the samples during the incubation period (pictures corresponding to 7 and 19 days of composting). At 28 days, clear deterioration of the samples is observed. The specimens are eroded and broken. As it has been reported in the literature, the biodisintegration process of PHB occurs by erosion from the surface to the bulk being the amorphous regions degraded in first place [55,56]. This leads to embrittlement and breakage of the samples. At 33 days of composting, all the samples appear broken into very small fragments. 

According to these results, it can be concluded that neither the fibers nor the reactive agents have a negative effect on the biodisintegrability of PHB in composting conditions. 

## 4. Discussion

Analyzing together the results obtained for the composites prepared with TC90, U_RH, and T_RH fibers with and without HMDI, it seems clear that the compatibility efficiency of the HMDI depends to a large extent on the purity of the fibers. Probably, this dependence is due to a greater presence of OH groups on the surface of the purified fibers, since it is assumed that compatibilization occurs through the formation of urethane bonds between the isocyanate groups of the HMDI and the hydroxyl groups of the fibers [33]. Tran et al. [57] also showed better results in PLA composites filled with rice and einkorn wheat husks using silane coupling agents, when fibers were previously submitted to an alkaline treatment. 

As reported in previous works [54], U_RH fibers present waxes and impurities on their surface, which could be responsible for their poor adhesion with the PHB matrix. In addition, in lignocellulosic fibers, the cellulose is embedded in a matrix of non-cellulosic components (mainly composed by lignin, hemicelluloses, and pectin). All this limits the exposition of the reactive –OH groups of the cellulose for compatibilization. The purification treatment applied removed those waxes and impurities on the fibers surface, as well as most of the non-cellulosic components, leaving a greater amount of reactive –OH groups at the surface that could react with the compatibilizer. In addition, the treatment resulted in fibers with more favorable morphology and surface characteristics (i.e., a higher aspect ratio and roughness) that also contributed to improve the mechanical properties, even without the presence of a compatibilizer.

## 5. Conclusions

In this work, PHB/TC90 composites were obtained by reactive extrusion using four different reactive agents: dicumyl peroxide (DCP), hexamethylene diisocyanate (HMDI), resorcinol diglycidyl ether (RDGE), and triglycidyl isocyanurate (TGIC). The effect of aging attending on the influence of the reactive agents and the fibers on the mechanical and crystallization behavior of the composites was assessed. Aging produced a drastic embrittlement of the PHB characterized by increased elastic modulus and decreased tensile strength, elongation at break, and toughness. This embrittlement was attributed to secondary crystallization, which increased the value of X_c_ by about 17% in neat PHB samples, but also to the physical aging of the amorphous fraction. Both fibers and reactive agents partly hinder secondary crystallization, leading to lower crystallinity in aged composites with respect to neat PHB. The melting temperature and crystallinity index for the compositions containing DCP were reduced at high cooling rates (processing conditions) and low cooling rates (DSC conditions), suggesting crosslinking of the PHB matrix. 

No clear compatibilization effect was found for the reactive agents DCP, RDGE, and TGIC. Elastic modulus, tensile strength, and elongation at break remained practically unchanged for PHB/TC90/DCP and PHB/TC90/TGIC compared with PHB/TC90. In the case of PHB/TC90/RDGE, those values were even lower than PHB/TC90. On the contrary, the tensile strength in PHB/TC90/HMDI was improved by about 40% with respect to neat PHB and around 18% with respect to the PHB/TC90 composite. The improved mechanical performance together with the SEM observations indicate enhanced interfacial adhesion between the TC90 fibers and PHB. 

A clear influence of the purity of the fibers on the effectiveness of HMDI at improving interfacial adhesion was observed. HMDI only demonstrated effectiveness in purified cellulose fiber composites, the greatest improvements in the mechanical performance being the ones obtained with highly purified commercial cellulose fibers (TC90). 

The different reactive agents did not negatively affect the compostability of PHB. 

Finally, thermoformed trays with good mold reproducibility were successfully obtained for PHB/T_RH/HMDI composite.

## Figures and Tables

**Table 1:** Summary of studied formulations. DCP: dicumyl peroxide, HMDI: hexamethylene diisocyanate, RDGE: resorcinol diglycidyl ether, TGIC: triglycidyl isocyanurate.

Sample		Component (phr)						
	PHB	TC90	U_RH	T_RH	DCP	HMDI	RDGE	TGIC
PHB	100			-	-	-	-	-
PHB/DCP	100			-	1	-	-	-
PHB/HMDI	100			-		1	-	-
PHB/RDGE	100			-	-	-	1	-
PHB/TGIC	100			-	-	-	-	1
PHB/TC90	100	10		-	-	-	-	-
PHB/TC90/DCP	100	10		-	1	-	-	-
PHB/TC90/HMDI	100	10		-	-	1	-	-
PHB/TC90/RDGE	100	10		-	-	-	1	-
PHB/TC90/TGIC	100	10		-	-	-	-	1
PHB/U_RH	100		10	-	-	-	-	-
PHB/U_RH/HMDI	100		10	-	-	-	-	-
PHB/T_RH	100			10	-	-	-	-
PHB/T_RH/HMDI	100			10	-	1	-	-

**Table 2:** Differential scanning calorimetry (DSC) parameters for neat PHB with and without reactive agents and PHB/TC90 composites with and without reactive agents.

	1st Heating Scan						Cooling Scan		2nd Heating Scan		
	0 days			100 days			T_c_ (°C)	ΔH_c_ (J/g)	T_m_ (°C)	ΔH_m_ (J/g)	X_c_ (%)
	T_m_ (°C)	ΔH_m_ (J/g)	X_c_ (%)	T_m_ (°C)	ΔH_m_ (J/g)	X_c_ (%)					
PHB	175	72	49	170	86	59	117	91	170	94	64
PHB/DCP	167	71	49	167	73	50	115	81	160	84	58
PHB/HMDI	173	69	48	173	79	55	106	86	167	90	62
PHB/RDGE	172	75	52	173	81	56	115	88	168	91	63
PHB/TGIC	173	77	53	172	80	56	116	89	168	92	64
PHB/TC90	173	74	56	173	73	55	117	82	169	84	63
PHB/TC90/DCP	166	67	51	165	66	50	118	75	161	78	59
PHB/TC90/HMDI	173	71	49	173	72	55	111	78	169	81	62
PHB/TC90/RDGE	172	76	52	173	73	56	115	79	169	85	65
PHB/TC90/TGIC	167	78	54	172	73	55	118	79	166	85	64

**Table 3:** Mechanical parameters corresponding to tensile tests.

	0 Days	15 Days	0 Days	15 Days	0 Days	15 Days	0 Days	15 Days
	Elastic Modulus (GPa)		Tensile Strength (MPa)		Elongation at Break (%)		Static Toughness (mJ/m^3^)	
PHB	1.41	3.04	25.89	23.03	4.96	0.98	0.84	0.13
P309/DCP	1.54	2.75	24.88	29.77	3.87	1.55	0.62	0.27
P309/HMDI	1.82	3.15	23.36	24.36	2.50	0.86	0.38	0.10
P309/RDGE	1.54	2.90	23.42	29.32	3.62	1.55	0.57	0.27
P309/TGIC	1.48	3.03	24.76	30.95	4.28	1.43	0.72	0.25
P309/TC90	1.71	3.44	21.27	27.14	3.49	1.21	0.53	0.19
P309/TC90/DCP	1.92	3.19	23.64	26.83	2.23	1.15	0.34	0.18
P309/TC90/HMDI	2.31	3.49	26.75	32.11	2.04	1.16	0.35	0.20
P309/TC90/RDGE	2.10	3.40	21.71	25.06	2.42	1.07	0.37	0.16
P309/TC90/TGIC	2.02	3.59	23.89	28.06	3.22	1.22	0.56	0.21
